# What do we know about gliotransmitter release from astrocytes?

**DOI:** 10.1098/rstb.2013.0592

**Published:** 2014-10-19

**Authors:** Daniela A. Sahlender, Iaroslav Savtchouk, Andrea Volterra

**Affiliations:** Department of Fundamental Neurosciences, University of Lausanne, Rue du Bugnon 9, Lausanne 1005, Switzerland

**Keywords:** gliotransmission, regulated exocytosis, channels, astrocytes, glutamate, electron microscopy

## Abstract

Astrocytes participate in information processing by actively modulating synaptic properties via gliotransmitter release. Various mechanisms of astrocytic release have been reported, including release from storage organelles via exocytosis and release from the cytosol via plasma membrane ion channels and pumps. It is still not fully clear which mechanisms operate under which conditions, but some of them, being Ca^2+^-regulated, may be physiologically relevant. The properties of Ca^2+^-dependent transmitter release via exocytosis or via ion channels are different and expected to produce different extracellular transmitter concentrations over time and to have distinct functional consequences. The molecular aspects of these two release pathways are still under active investigation. Here, we discuss the existing morphological and functional evidence in support of either of them. Transgenic mouse models, specific antagonists and localization studies have provided insight into regulated exocytosis, albeit not in a systematic fashion. Even more remains to be uncovered about the details of channel-mediated release. Better functional tools and improved ultrastructural approaches are needed in order fully to define specific modalities and effects of astrocytic gliotransmitter release pathways.

## 1. Introduction

The importance of astrocytes in brain function is increasingly appreciated. In addition to providing homeostatic control and metabolic support, they play an active role in information processing within neuronal circuits [1,2]. As part of the bi-directional communication, astrocytes can both receive and send chemical signals to neurons. Numerous studies have shown that astrocytes can respond to various neurotransmitters and factors and in turn release glutamate, d-serine, ATP, and gamma-aminobutyric acid (GABA) as well as prostaglandins and neuropeptides in a process termed gliotransmission (for some recent reviews, see [3,4]).

Various mechanisms have been proposed for such gliotransmission, including Ca^2+^-regulated vesicular exocytosis and non-vesicular release. Non-vesicular release may be achieved via plasma membrane ion channels, such as connexin/pannexin hemichannels, purinergic P2X7 channels, volume-regulated anion channels and via pumps, such as the glutamate–cysteine exchanger or the glutamate uptake system working in reverse [3]. So far, there is no clear evidence that channel-mediated (connexins/pannexins/P2X7) mechanisms are relevant to physiological communication between astrocytes and neurons, because opening of the channels was shown to occur mainly under non-physiological conditions such as traumatic injury, seizure activity and altered oxygen and glucose concentrations [5,6]. In view of this, Ca^2+^-dependent exocytosis has been proposed as the major mechanism for release of the main gliotransmitters (glutamate, d-serine and ATP), similar to what happens in neurons and secretory cells. More recently, however, an anion channel, Bestrophin-1 (Best-1), has been reported to play a role in GABA and glutamate release from astrocytes under physiological conditions [7,8]. Furthermore, a very fast (milliseconds) but Ca^2+^-independent release of glutamate was reported via the potassium channel TREK-1 [8]. It is, however, presently not clear how a channel structurally devised to selectively flux a small cation such as K^+^ can adapt to release a negatively charged and much bigger organic anion such as glutamate. In conclusion, whether regulated exocytosis or a channel-mediated mechanism (or both) is responsible for gliotransmission under physiological conditions presently remains a topic of active debate [3,9].

Neither the molecular mechanisms of gliotransmitter release, nor their regulation are yet fully understood. However, Ca^2+^ is believed to be the main regulator of gliotransmission. Transient rises in intracellular Ca^2+^ concentration have been observed upon astrocyte stimulation *in situ* and *in vivo* and often shown to be important for gliotransmitter release. For some recent reviews on the role and sources of Ca^2+^ in astrocytes, see e.g. [1,10], but also see [11,12]. In this review, we shall focus on the downstream effects of Ca^2+^ on gliotransmission. In particular, we will discuss vesicular exocytosis and Best-1 channel release because both these pathways are Ca^2+^-dependent and putatively physiologically relevant.

## 2. Regulated exocytosis and release via channels from astrocytes

**Figure 1.**
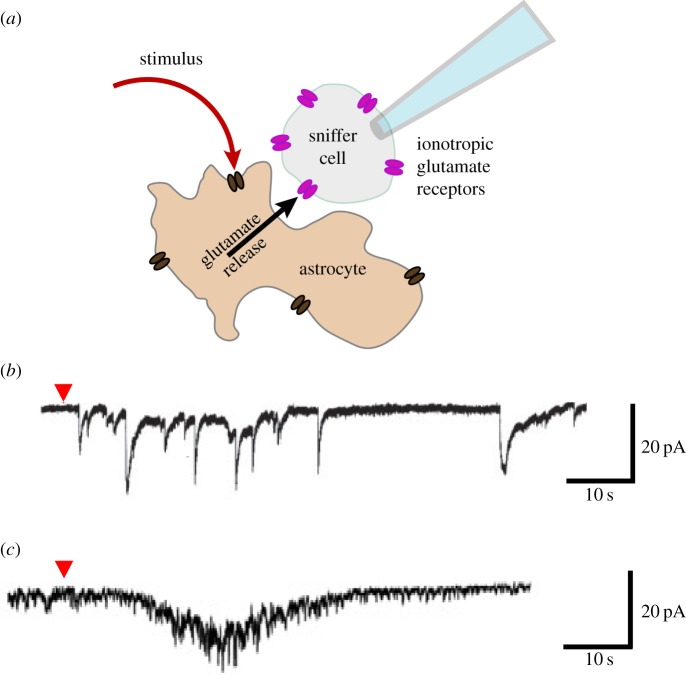
Two modes (vesicular and channel-mediated) of glutamate release detected by sniffer cells. (*a*) Schematic diagram of the sniffer cell experiments; astrocytes stimulated by receptor agonists; sniffer cells expressing transfected ionotropic glutamate receptors. (*b*) Astrocytic activation by the application of the AMPAR/mGluR agonist l-quisqualate (red arrowhead) triggers a rapid burst of excitatory post-synaptic currents (EPSCs) in an *N*-methyl-d-aspartate (NMDA) receptor-expressing HEK ‘sniffer’ cell. These EPSCs are thought to reflect quantal events of vesicular glutamate release from the astrocyte (adapted with permission from [13], fig. 3b). (*c*) Application of a TFLLR agonist of the PAR-1 metabotropic receptors (arrowhead) to astrocytes triggers a slow current in HEK cells expressing non-desensitizing α-amino-3-hydroxy-5-methyl-4-isoxazolepropionic acid (AMPA) receptors. This current is thought to be mediated by glutamate release through anion channel Best-1 and disappears in Best-1 knockout or knock-down astrocytes (adapted with permission from [8], sniffer experiments on a cultured astrocyte transfected with TREK-1 shRNA, fig. 2i).

Gliotransmitters may be released from a storage compartment via exocytosis, or directly from the cytosol via plasma membrane ion channels. In theory, release through membrane channels or transporters would be energetically ‘cheaper’ than transporting and pre-concentrating a transmitter inside a secretory compartment, often against a steeper electrochemical gradient. However, the nature of channel release has other limitations, namely that a large amount of transmitter cannot be released all at once (as it can be by pre-concentrating it inside a vesicle). Instead, smaller amounts of transmitter may be released per unit time, but on a much longer timescale. As such, temporal precision and maximum peak concentrations are sacrificed. The two mechanisms may, therefore, have different functional consequences (see figure 1).

Regulated exocytosis in neurons is triggered when an action potential (AP) reaches the nerve terminals and evokes a local Ca^2+^ increase that, in turn, triggers rapid (less than a millisecond) soluble NSF attachment protein (SNAP) receptor (SNARE)-dependent fusion of release-ready synaptic vesicles (SVs) with the plasma membrane. AP-dependent synaptic release relies on the interaction of the SNARE proteins, VAMP2, Syntaxin 1 and SNAP25, as well as other regulatory proteins including the Ca^2+^ sensor, such as synaptotagmin I [14]. In astrocytes, earlier studies in cultures have suggested that they likewise express VAMP2, raising the question of whether astrocytes are also competent for regulated exocytosis [15].

Release from the cytosol via plasma membrane ion channels does not directly require a SNARE-mediated fusion event. However, channel trafficking and insertion into the plasma membrane may still involve Ca^2+^ changes and SNARE proteins, albeit in general on a much longer timescale compared with regulated exocytosis. It can take hundreds of seconds to insert a channel into the membrane after a stimulus (see e.g. AMPA receptors [16]).

### (a) Functional evidence for regulated vesicular exocytosis in astrocytes

To examine whether regulated exocytosis is the mechanism underlying the modulatory actions of astrocytes in synaptic function and plasticity, several approaches have been taken. First, the release was shown to be Ca^2+^-dependent, because infusion of Ca^2+^ buffer solutions in astrocytes led to perturbation of synaptic activity [17–20]. However, this approach *per se* is insufficient to identify exocytosis as the mechanism of the release because channel opening can also be controlled by Ca^2+^ [8,21]. Therefore, the mechanism was probed further using tetanus or botulinum toxins that cleave VAMP2/3 or SNAP 23/25 selectively, and which were shown to block regulated exocytosis in neurons [22]. By putting the membrane-impermeant light chain fragment of tetanus toxin (TeNT_LC_) inside astrocytes, the synaptic effects of gliotransmission were blocked [17,19,23,24]. Additionally, two mouse models, dubbed ‘dnSNARE’ and ‘iBot’, were generated to interfere specifically with VAMP2 and 3 in astrocytes (see box 1). Use of the above mice has generally resulted in the perturbation of synaptic properties of the neuronal circuit and suggested that VAMP2 or 3-dependent exocytosis from astrocytes was involved.

Box 1. **Genetic mouse models to study regulated exocytosis from astrocytes.**

So far the use of genetic mouse models for testing the physiological relevance of Ca^2+^-dependent exocytosis from astrocytes has been aimed at interfering with SNARE proteins required for regulated exocytosis. Two models were created, namely the dominant negative (dn)SNARE mouse, which overexpresses VAMP2 lacking its transmembrane domain in GFAP-positive cells in a doxycycline-inducible manner [25], and the ‘iBot’ mouse, which expresses clostridial botulinum neurotoxin serotype B light chain (BoNT/B) in GLAST-positive astrocytes using the inducible Cre/loxP system [26]. The dnSNARE mouse has the goal to prevent VAMP2-dependent exocytosis specifically in astrocytes by outcompeting endogenous VAMP2, and hence to block VAMP2-dependent fusion events. Similarly, the ‘iBot’ mouse is meant to prevent exocytosis by overexpressing specifically in astrocytes active BoNT/B which cleaves and inactivates VAMPs 2 and 3 [22,27]. It should be noted that while VAMP2 has been described as being present in astrocytic cultures [15,28], it appears to be low or absent in the adult hippocampal astrocytes *in situ* [29]. Instead, astrocytes express VAMP3 at high levels [29–31]. It is known that VAMPs can participate in the formation of several different SNARE complexes by pairing with more than one set of partners [32]. In view of this, VAMP3-dependent fusion is still likely to be disrupted in the dnSNARE mouse. However, because VAMP3 is also involved in constitutive protein trafficking, it is unclear whether other VAMP3-dependent fusion events would be affected in the dnSNARE mouse. Moreover, since the dnSNARE mouse is activated by doxycycline treatment over several weeks, compensatory mechanisms may occur.

To date, the dnSNARE mouse has been used in multiple studies, providing the important demonstration that SNARE-dependent pathway(s) in astrocytes are critically involved in several physiological and pathological processes, including sleep, inflammation, drug-seeking behaviour or stroke [33–35]. While this mouse has been used for *in vivo* and behavioural studies, it has not fully provided detailed mechanistic insight so far. For example, biosensor studies have suggested that overexpression of the dnSNARE domain results in changes in extracellular adenosine levels [36] but have been less clear concerning extracellular glutamate or d-serine [37]. This is possibly due to the low sensitivity of the biosensor approach *in situ* or *in vivo*, particularly with respect to extracellular glutamate detection. Indeed, strong expression of high-affinity glutamate transporters in the tissue makes likely that any released glutamate is rapidly taken up before being detected by the sensor, unless the location of release is targeted precisely.

Similar concerns have been raised regarding the newer ‘iBot’ mouse model, which has not been as widely used to date. Here, both VAMP2 and VAMP3 should be inactivated by the protease activity of the toxin, but it is not clear how efficiently this occurs in astrocytes *in situ*. In particular, it is not known whether sufficient levels of the toxin are expressed in all astrocytes, and whether the toxin is active over time. Unfortunately, the crucial experiment designed to show whether VAMP2 and 3 are cleaved efficiently in astrocytes and retinal Mueller cells *in vivo* [26] is not clear since the protease activity was not properly demonstrated. As with the dnSNARE mouse, specificity can be also an issue, because the cytoplasmic expression of BoNT/B will target all VAMP3-dependent fusion events, including possibly constitutive exocytosis.

However, dependency of the release on SNAREs and Ca^2+^ is still not sufficient proof that gliotransmitter release is achieved via regulated exocytosis because SNARE proteins and Ca^2+^ may be equally important for insertion of plasma membrane-bound ion channels (such as Best-1) or pumps [3], although the latter process is much slower. Therefore, to investigate the mechanism further, attempts were made to interfere with other aspects critical for exocytic release such as vesicular uptake of transmitter or to determine the speed of release. Here, the results obtained for the main gliotransmitters, d-serine, ATP and glutamate are summarized:

#### (i) d-serine

The release of d-serine from astrocytes in the CA1 region of the hippocampus was shown to be important for long-term potentiation (LTP) at Schaffer collateral–pyramidal cell synapses. Experiments indicated that this form of LTP is disrupted by selective infusion of astrocytes with TeNT_LC_ or ‘Ca^2+^ clamp’ solution [19] (but see [38]). Importantly, by blocking d-serine production inside the astrocyte with the serine racemace inhibitor HOAsp, it was demonstrated that d-serine can nevertheless remain stored for prolonged periods in a putative astrocytic compartment. Moreover, this d-serine pool was depleted by pre-stimulation experiments, leading to the abolition of LTP [19]. These observations support a vesicular rather than a channel-mediated mode of d-serine release from astrocytes during LTP induction.

#### (ii) ATP

The dnSNARE mouse model was used extensively to investigate ATP release from astrocytes [25]. In several studies using this model, ATP release was generally reduced, but temporal information was hard to obtain (see details in box 1). However, a recent study was able to observe exocytosis of astrocytic ATP in the cortex directly, by monitoring quantal release onto P2X4 neuronal receptors [39]. ATP release was detected within hundreds of milliseconds from astrocytic activation and corresponding Ca^2+^ increase. The detected ATP events were quantally distributed, suggesting that a storage compartment is involved [39].

#### (iii) Glutamate

One approach to study glutamate release from astrocytes has focused on the presence and function of vesicular glutamate transporters (vGluts) in these cells. Currently available vGlut antagonists (e.g. Evans Blue and Rose Bengal) are poorly selective. Nevertheless, experiments using these agents in tissue have produced intriguing results. Infusing the astrocytes with Evans Blue has abolished spike-timing-dependent long-term depression (LTD) at nearby synapses in the cortex [24]. The same effect was obtained by patching the astrocytes with a ‘Ca^2+^ clamp’ or with TeNT_LC_. All these results are consistent with a vesicular nature of the astrocytic release [24], at least in this preparation.

In the hippocampal dentate gyrus, glutamate release from astrocytes was shown to activate pre-synaptic NMDARs and thereby increase transmitter release probability at nearby perforant path–granule cell synapses [20,23]. Astrocytes were stimulated electrically and the changes in synaptic release (e.g. changes in miniature (m)EPSC frequency) were monitored [23]. The increase in synaptic release probability occurred within seconds of astrocytic stimulation and was blocked by prior infusion of astrocytes with the Ca^2+^ chelator BAPTA [20,23] or with TeNT_LC_ but not of a non-functional mutant version of the toxin. Because the astrocyte-evoked synaptic effect was fast (approximately in seconds) and Ca^2+^ and SNARE dependent [23], it was concluded that glutamate was released by regulated exocytosis in this system. Similarly, fast (approx. 10 s) modulation of neuronal release by astrocytic glutamate was observed at immature GABAergic synapses [40,41].

### (b) Functional evidence for channel-mediated gliotransmitter release

Besides regulated exocytosis, gliotransmitter release may occur directly from the cytosol by flux across plasma membrane ion channels. Regulated release through membrane channels in astrocytes is possible because these cells are known to express a large variety of anion channels (for reviews, see e.g. [42]), including Ca^2+^-dependent Cl^−^ channels [43]. Anion channels generally tend to be promiscuous, allowing flux of both small inorganic anions such as Cl^−^, as well as larger anions such as hydrocarbonate, GABA, glutamate, taurine and l-aspartate. For example, release of taurine from astrocytes via volume-regulated anion channels plays a significant physiological role in modulating neuronal firing by releasing taurine in osmosensitive areas of the hypothalamus [44]. Release of glutamate via Ca^2+^-regulated channels was reported in tissue several years ago, although the molecular identity of the anion channel was not established at that time [21]. More recently, the anion channel Best-1 has been shown to function in GABA release from cerebellar glia [7] and in glutamate release from astrocytes in the CA1 of the hippocampus [8,45]. What are the properties of glutamate and GABA release via a channel?

Best-1 conductance was shown to be Ca^2+^-dependent by patch-clamping cells using internal solutions containing a range of free Ca^2+^ concentrations, but its conductance can also be stretch (osmolarity) dependent [46]. Whether Best-1 directly senses Ca^2+^ or is modulated by an intermediary Ca^2+^-sensitive protein is unclear. It has been proposed that Best-1 can directly sense Ca^2+^ via a Ca^2+^-binding domain that can modulate channel opening [47]. A separate form of Best-1 regulation is via phosphorylation [48].

In a culture system or in acutely dissociated astrocytes, it has been shown that glutamate release through Best-1 is relatively slow. The delay between astrocytic stimulation (by puffing an agonist of the protease activator receptor PAR-1) and the resulting glutamate detection in sniffer cells expressing an engineered AMPA receptor is in the order of 10–20 s [8]. By contrast, in a comparable system, regulated exocytosis in astrocytes can be much more rapid. In culture, astrocytic vGlut2-GFP- or vGlut1-pHluorin-positive vesicles were reported to fuse within tens of milliseconds of receiving a stimulus, such as a G protein-coupled receptor (GPCR) agonist [30,49], which is nearly three orders of magnitude faster than the Best-1 channel-mediated release.

In a recent study, it was concluded that Best-1-mediated glutamate release is TeNT insensitive [8]. This was despite a reported reduction in the sniffer cell AMPA receptor currents within 20 min of the astrocytes being patched with a TeNT-containing solution. This reduction was attributed to a ‘side-effect’ of the toxin, exiting the astrocyte and inhibiting the sniffer cell receptor expression on the cell surface [8]. However, it is not clear whether the holotoxin TeNT (which can cross membranes) or TeNT_LC_ (which cannot cross membranes) was used in these experiments. In keeping, a prior report of glutamate release through an anion channel which used an enzymatic assay rather than the sniffer cell assay, showed no sensitivity to TeNT even after 24 h incubation with the toxin [21]. These data would suggest that the turnover of Best-1 insertion at the plasma membrane is extremely slow.

### (c) Morphological evidence for gliotransmitter release from a storage compartment

In contrast to the ion channel-mediated mode of release for which channels should be localized at the plasma membrane, Ca^2+^-regulated exocytosis relies on a dedicated compartment that stores gliotransmitters. This needs to be equipped with a transporter that pumps the gliotransmitter into the compartment, as well as with the proteins that mediate and regulate the fusion event. There is no doubt that astrocytes contain tubular-vesicular structures as part of the endocytic and exocytic pathway (see box 2) but is there a compartment competent for regulated exocytosis of gliotransmitters?

Box 2. **EM analysis of astrocytes.**

**Figure 2.**
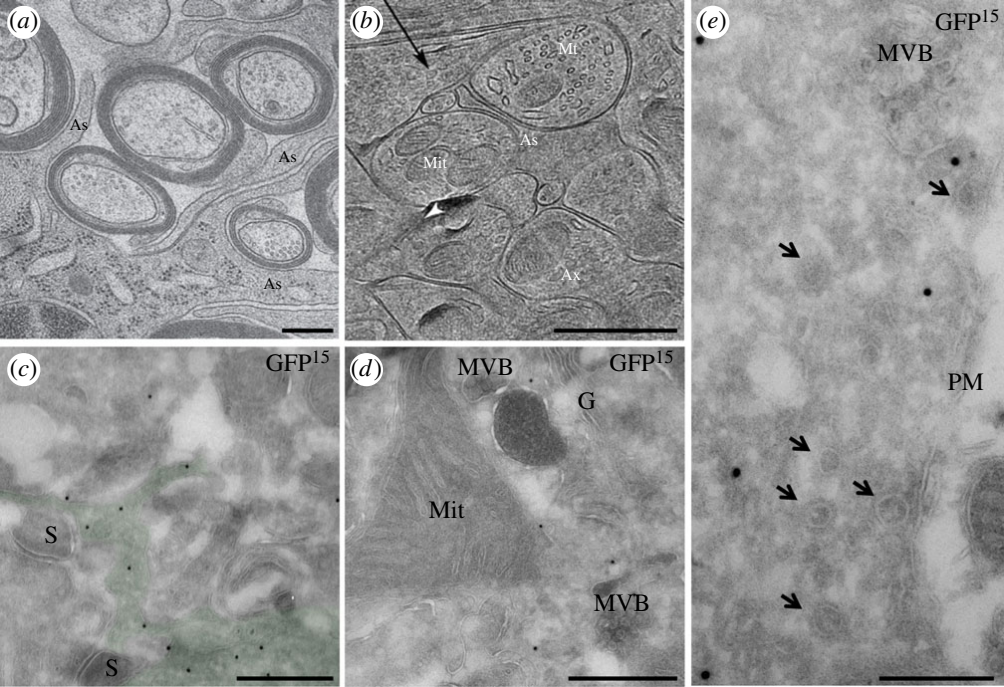
Different EM techniques reveal organelles and the dense cytoplasm of astrocytes in the tissue. (*a*) Using a HPF/FS preparation, it has been shown that astrocytic processes have a dense and homogeneous cytoplasm. Adapted with permission from [54], (*b*) Cryo-EM analysis of thin sections of high-pressure frozen organotypic brain slices shows that astrocytic perisynaptic processes have a dense cytoplasm indistinguishable from the cytoplasm of neurons. Adapted with permission from [55], copyright 2005 National Academy of Sciences, USA, (*c*–*e*) Tokuyasu preparation of the GFAP-eGFP mouse [56] labelled with anti-GFP and 15 nm gold particles (D.A.S. and A.V. 2012, unpublished data). Also in this preparation, the astrocytic cytoplasm is dense and organelles are readily visible; (*c*) small astrocytic processes surrounding synapses (S) display dense cytoplasm (astrocytic structures are identified by the presence of GFP and have been highlighted in green); (*d*) organelles are present in the astrocytic cytoplasm; (*e*) tubular-vesicular structures (indicated by arrows) are present close to the plasma membrane (PM). As, astrocyte; Ax, axon; Mit, mitochondria; G, golgi complex; MVB, multivesicular body; scale bars: 500 nm. (Online version in colour.)

EM has been an essential tool to establish the existence and organization of SVs in neurons. Much less is known about the organization and distribution of SLMVs in astrocytes in the tissue and about the distribution of astrocytic organelles in general. Whereas SVs can be readily identified due to their accumulation in the pre-synapse, SLMVs may be more difficult to find (see §2c). So far, research has focused on the astrocytic processes that enwrap synapses, from which gliotransmitters are thought to be released [30]. However, some investigations have suggested that these perisynaptic processes are devoid of endoplasmic reticulum [50] and sparse in organelles in general (reviewed in [51]), which would question the idea that they support Ca^2+^-regulated exocytosis and gliotransmission [50]. Such a negative conclusion needs to be taken cautiously. Indeed, almost no study to date has performed a systematic and quantitative ultrastructural analysis of astrocytes, of the distribution of organelles within astrocytes and of the relationship between astrocytic and neuronal structures. Moreover, and crucially, good preservation of astrocytes in preparations of the rodent brain using conventional EM methods has not been sufficiently considered. Indeed, most studies to date have shown that the cytoplasm of astrocytes is electron lucent (‘empty’), and this feature has even been used as a criterion to identify and distinguish astrocytes from neurons in the neuropil [52,53]. It should be noted, however, that astrocytes and their fine processes do not always appear empty (see figure 2). As discussed many years ago by Peters *et al*. [57], astrocytes are certainly not electron lucent but this may be the result of suboptimal tissue preparation. Astrocytes are in contact through their end-feet with blood vessels and therefore may be prone to volume changes in poorly fixed preparations [57], which could affect the cytoplasm as well as the distribution of intracellular organelles. Although the majority of EM studies so far have relied on conventional EM techniques, including chemical fixation and resin embedding, when other preparations such as high-pressure freezing (HPF) and freeze substitution (FS) [54] (figure 2*a*), CEMOVIS [55] (figure 2*b*) or the Tokuyasu method [58] (figure 2*c*–*e*) are used, the appearance of the astrocytic cytoplasm is similar to that of other cells and organelles can be readily identified, including in the perisynaptic processes. HPF and FS techniques have been shown in particular to be advantageous in better preserving the morphology of cells within tissue, including the extracellular space [54]. There may be less fixation-induced osmotic shock and less cell swelling or shrinkage with this method. Another common EM technique used in cell biology is the Tokuyasu preparation, which has not been routinely used for brain tissue. The major difference is that the samples are neither fully dehydrated nor embedded in a resin after fixation and that antigenicity can be preserved. These approaches, together with additional new technologies, including focused ion beam scanning EM and serial block-face scanning EM will help to analyse astrocytes in three dimensions in large tissue volumes more systematically [59,60] and are expected to provide more advanced and reliable information on their ultrastructural characteristics.

In astrocytic cultures, several compartments have been shown to undergo regulated exocytosis, including a vesicular compartment termed small synaptic-like microvesicles (SLMVs), secretory lysosomes and dense core granules [30,61,62]. For example, ATP has been shown to be released from secretory lysosomes [63,64]. However, different methods used to load organelles with dyes in culture have produced ambiguous results [65]. Based on electron microscopy (EM) analysis of astrocytes in rodent brain tissue, SLMVs have been suggested as the main storage compartment for gliotransmitters, including glutamate and d-serine [66]. In tissue, SLMVs range from 30 to 80 nm in diameter and are often found in close proximity to the plasma membrane in the perisynaptic processes of astrocytes [23,30,66]. Whether other organelles, such as secretory lysosomes and dense core granules are involved in gliotransmitter release *in situ* has not been thoroughly investigated yet.

To further characterize SLMVs, they have been isolated from astrocytic cultures and investigated biochemically [67,68]. Recent comparison of isolated SLMVs and neuronal SVs has suggested that astrocytic SLMVs differ from SVs in protein composition and transmitter content as well as in the mechanism of transmitter loading [68]. Whereas isolated SVs contained typical synaptic markers, such as synaptotagmin I and synaptophysin, SLMVs were negative for those but positive for vGlut2, vesicle-associated membrane protein (VAMP)2 and VAMP3 as shown earlier [67,68]. The investigation of isolated SLMVs has further suggested that they can store d-serine and glutamate in the same vesicle and co-release them [68]. By contrast, SVs contained glutamate, glycine and GABA but were devoid of d-serine. Whether SLMVs *in vivo* can co-release d-serine and glutamate and whether this has specific functional implications is still unclear as immuno-EM studies have tentatively attributed the presence of d-serine and glutamate to distinct SLMVs populations within the same astrocyte [66].

### (d) Proteins involved in regulated exocytosis in astrocytes

Initial studies in cultured astrocytes have found that these cells express VAMP2 in addition to VAMP3 [15], which have focused attention on isolating VAMP2-containing vesicles [67,68]. However, more recent immunohistochemistry studies in tissue have shown that VAMP2 may be weakly expressed or even not present in mature astrocytes in the hippocampus [29]. Consistently, the protein was not found on SLMVs by immuno-EM [30]. Instead, its ubiquitously expressed homologue VAMP3 has been detected in astrocytes [29,30]. Interestingly, VAMP3 has been localized to vGlut1-positive SLMVs in the tissue by EM [30], suggesting that VAMP3 is important for regulated exocytosis of SLMVs. This is also consistent with the functional data obtained with TeNT_LC_ which does not discriminate between VAMP2 and VAMP3. Furthermore, it has been shown that putative SNARE partners in astrocytes include SNAP23 but not SNAP25 [15,29,31]. Ca^2+^ sensitivity of exocytosis is provided by a Ca^2+^ sensor protein, whose identity in astrocytes is not yet known. However, mRNA studies in the tissue have detected co-expression of several synaptotagmin isoforms in astrocytes, including either synaptotagmin 7 and 11 or synaptotagmin 1 and 4 [69]. Synaptotagmin 4 has been reported to be present on isolated astrocytic vesicles and proposed to be involved in regulated exocytosis [67,70], although the Ca^2+^ sensitivity of synaptotagmin 4 is debated [71].

### (e) Vesicular transporters found in astrocytes

Transmitter release from a storage compartment requires a vesicular transport system. In general, astrocytes are thought to be unable to maintain a high rate of transmitter release as observed during tetanic trains in neurons due to slower stimulus–secretion coupling. Therefore, a large reserve pool is not needed in these cells, and a small number of vesicles (approx. five) per release site may be sufficient. As such, the transporter levels in astrocytes will be low. The vesicular transporter for d-serine is not yet known, although a d-serine transport activity has been recently identified by functional studies [68]. The transporter for ATP, VNUT, is expressed in various cell types including astrocytes where it is important for release of ATP [39], although the identity of the compartment in which the transporter is located *in situ* is not clearly established. With regard to glutamate, its transporters vGluts1–3 are known to be highly expressed in neurons. However, astrocytic expression is reported to be lower, which has led to a debate whether vGluts are functionally relevant for glutamate release from astrocytes. Unlike in neurons, where vGluts are found on all SVs at glutamatergic terminals, including the large reserve pool (perhaps 100–1000s vesicles per each synapse), no such accumulation of vesicles is expected or has been reported in astrocytes so far. Instead, clusters of SLMVs containing 2–15 vesicles each were observed by EM [66], with a portion of them positive for vGluts [52]. The presence of vGluts in astrocytes has been investigated in several studies with various experimental approaches. Endogenous vGlut was detected by Western blotting or immunohistochemistry in astrocytic cultures, although the staining pattern varied between preparations [15,28,49,72]. Isolated vesicles have been shown to be positive for vGlut2 [67,68]. Various groups have localized vGluts in tissue, focusing on its astrocytic labelling with opposite results, either observing vGluts in astrocytes [23,28,30,52,66,73] or failing to do so [74–76]. VGlut1 was shown by both light microscopy and EM to be present in astrocytes in various brain regions [52], although expression of the transporter in the mouse tissue could be quite low [52]. This could explain the negative results of some localization studies relying purely on light microscopy but not EM observations [74]. A problem with light microscopy is that the vGlut signal in astrocytes may not result in such a distinct staining pattern as observed in neurons due to a much smaller number of vesicles and to a different distribution in comparison to glutamatergic synapses. Moreover, by light microscopy, attribution of a protein to a distinct cell (astrocyte versus neuron) in the tissue is challenging if the protein is located close to the plasma membrane. Other sets of studies looked at the presence of vGlut transcripts in astrocytes. This was done, for example, by single-cell reverse transcriptase PCR. In the adult hippocampus, mRNA transcripts for either vGlut1 or 2 were detected in subpopulations of S100beta-positive astrocytes [30]. Gene chip microarray (transcriptome) analysis of purified astrocytes produced mixed results. One study reported only very low levels of vGlut transcripts from the entire forebrain of young mice [77], hence questioning the existence of glutamate gliotransmission, whereas others reported higher levels depending on the brain region [78]. These results highlight the importance of investigating astrocytes in different anatomical locations and underscore the heterogeneity of astrocytes due to location, age and species.

### (f) Localization of ion channels releasing gliotransmitters

Fewer studies have focused on the distribution of ion channels like Best-1 in different brain regions as well as at the cellular level. Best-1 is highly expressed in retinal epithelial cells and at lower levels in skeletal muscle, as well as kidney, colon and brain [79]. In the brain, Best-1 appears to be expressed in both neurons and astrocytes [7,80]. In the hippocampus, Best-1 labelling has also been investigated by EM [8]. The authors reported that Best-1 is mainly present at the plasma membrane of the perisynaptic processes that enwrap synapses, although they also observed an intracellular pool [8]. These are intriguing results, but they still require confirmation of the specificity of the Best-1 staining. Indeed, given that a Best-1 knockout (KO) mouse is available, the antibodies should be validated on the KO tissue.

## 3. A debated issue: are glutamate levels in astrocytes too low to support gliotransmission?

Different from neurons, astrocytes express the cytosolic enzyme glutamine synthetase (GS), which transforms glutamate into glutamine, therefore reducing the free cytosolic glutamate level. While there is consensus that cytosolic glutamate in tissue astrocytes is lower than in neurons, no reliable measure of its real concentration exists. It has been proposed that this reaches ‘housekeeping levels’ and hence it is too low to permit effective glutamate release from astrocytes ([81], critically reviewed in [2]), particularly if glutamate would be released from the cytosol via plasma membrane channels. To verify this hypothesis, theoretical calculations were made based on the proportion of immunogold particles labelling l-glutamate in the cytosol of astrocytes compared with adjacent nerve terminals in the tissue. According to such calculations, intracellular glutamate in astrocytes should be sufficient to be taken up by vesicles and exocytosed at concentrations able to activate high-affinity neuronal receptors such as NMDA receptors [3], even in the ‘worst case’ steady-state conditions. In practice, the situation could be more favourable: first, because, once taken up into vesicles, glutamate is protected from conversion to glutamine by GS and can be pre-concentrated to relatively high levels; second, because the interplay between uptake of extracellular glutamate, GS activity, pH changes and GS and vGluts affinities would favour vesicular uptake. Indeed, conversion of glutamate to glutamine by GS is pH sensitive and is reduced at lower intracellular pH [82]. At the same time, glutamate uptake into astrocytes is known to lead to local decrease of intracellular pH, likely by co-transport of H^+^ [83,84]. As a consequence, GS activity would be slowed down or suppressed [85] and if the local pH decrease occurs in a sub-membrane domain containing vGlut-expressing vesicles, as is likely, it is expected to favour vesicular uptake over conversion of glutamate to glutamine. This uptake is also favoured because the affinity of vGluts for glutamate (*K*_m_ = 1–3 mM [86]) is somewhat higher than that of GS (approx. 3 mM [87]). In conclusion, the above considerations, as well as the functional data showing modulatory actions of astrocytic glutamate on synaptic activity, do not support the hypothesis that glutamate levels in astrocytes are too low for effective release of the amino acid.

## 4. Concluding remarks

What do we actually know about gliotransmitter release from astrocytes? There is clear evidence that astrocytes respond to synaptic signals with gliotransmitter release. Gliotransmission has been observed with multiple experimental approaches from various independent groups [10] and cannot just simply be an artefact [9].

Two mechanistically different release modes have been proposed for regulated gliotransmission under physiological conditions: vesicular exocytosis or release via plasma membrane ion channels. For both, the functional experiments suggest that Ca^2+^ is the key regulator. Functional data in tissue based on SNARE-specific toxins and transgenic models point towards regulated exocytosis as the main release mechanism. Morphological observations have suggested SLMVs as the gliotransmitter storage compartment. Vesicular transporters for glutamate have been localized to astrocytic SLMVs by EM. However, there is also evidence for release of GABA and glutamate from the cytosol through plasma membrane ion channels such as Best-1, although this is a fairly recent observation and needs to be further investigated and confirmed by several laboratories independently.

It is still debated whether astrocytes communicate with neurons focally or at longer distance via volume transmission. In support of focal release, both SLMVs and Best-1 channels have been observed in astrocytic processes juxtaposed to synapses [8,23,30]. In the peripheral nervous system, a single glial cell was shown to be able to monitor and modulate two adjacent synapses independently [88]. Alternatively, volume transmission relies on diffusion of a transmitter over distance, which requires the absence of high-affinity uptake systems or degrading enzymes. In view of this, d-serine and, to some extent, GABA would be plausible as volume gliotransmitters, whereas glutamate is less likely to be one. These questions are yet to be addressed experimentally.

One important debated issue is whether all astrocytes release the same gliotransmitter(s). At the moment, studies have been carried out in different brain regions, focusing on a single type of gliotransmitter at a time. If release of one gliotransmitter is detected, this does not preclude release (or even co-release) of another gliotransmitter at the same time, or under different conditions. In neurons, vesicular co-release of two neurotransmitters has been reported at the same synapse. For example, vGlut and vGAT can coexist at the cerebellar basket cell terminals [89]. Similarly, dopaminergic neurons in nucleus accumbens, but not in dorsal striatum, can co-release glutamate [90]. Whether this happens also in astrocytes is not known, although co-transport of glutamate and d-serine has been reported to occur in isolated SLMVs [68]. This further raises the question of region specificity of transporters in astrocytes. Finally, an ion channel, such as Best-1, could also co-release gliotransmitters, although this possibility has not been tested yet. More systematic studies in all brain regions should be carried out for all gliotransmitters.

Why is there still so much controversy with regard to gliotransmission and its underlying mechanisms? Certainly, experiments conducted in different brain regions, at different ages and using different protocols or even different species (mouse versus rat) could lead to inherent differences in results. In order to have a better understanding of gliotransmission, more rigorous experimental settings, more specific mouse models, including astrocyte-specific inducible knockouts, and more selective antagonists for vesicular transporters and channels are needed. To investigate the protein composition of astrocytic organelles, proteomic analysis could be a useful tool as has been shown for SVs [91].

In conclusion, much work is still required in order to understand when, why and how astrocytes release gliotransmitters and, by this process, participate in information processing in the brain.
